# Growth Rate of *Pseudomonas aeruginosa* Biofilms on Slippery Butyl Methacrylate-Co-Ethylene Dimethacrylate (BMA-EDMA), Glass and Polycarbonate Surfaces

**DOI:** 10.4172/2155-952X.1000274

**Published:** 2017-11-02

**Authors:** Helena Valquier-Flynn, Christina L Wilson, Andrea E Holmes, Christopher D Wentworth

**Affiliations:** 1 Department of Chemistry, Doane University, Nebraska, USA; 2 Department of Physics and Engineering, Doane University, Nebraska, USA

**Keywords:** Biofilm growth rate, Antimicrobial resistance, *Pseudomonas aeruginosa*, Porous polymer surface

## Abstract

Bacterial biofilms pose a significant health risk when they grow on devices placed or implanted in the human body. There is a need to develop new materials that can be used as surface coatings on such devices to inhibit biofilm growth. We report on measurements of the biofilm growth rate on a new polymeric material, slippery BMA-EDMA, which can be used as a surface coating for medical devices. Growth rate measurements are also reported for polycarbonate and glass surfaces, for comparison. Measurements are made in a medium shear stress fluid environment. The physical properties of the surfaces are characterized using contact angle, surface roughness, surface skewness and surface kurtosis. Growth rate on the slippery BMA-EDMA is found to be the smallest of the three surfaces. Growth rate is weakly correlated with surface hydrophobicity and surface roughness, while it is strongly correlated with surface skewness and kurtosis.

## Introduction

Biofilms are structured, matrix-enclosed microbial communities that adhere to a surface or interface and are now understood to be the most prevalent form taken by bacteria in natural, industrial and medical aquatic environments [1]. Many bacterial infections in humans involve biofilms growing on tissue, as in necrotizing fasciitis or on implanted devices including catheters, artificial heart valves and orthopedic devices [2]. Such biofilm infections are typically not resolved by host immune response or antimicrobial therapy and must be mechanically eliminated by surgery or device removal [3]. Developing methods of either preventing or disrupting biofilm growth on tissue or devices in the human body is of great current interest.

*Pseudomonas aeruginosa* is a common environmental bacterial species of class Bacillus, which acts as an opportunistic pathogen in immune compromised individuals and is involved in a broad spectrum of bacterial infections including infection of tissue in severe burn victims, acute lung infection in cystic-fibrosis patients, and ulcerative keratitis occurring in contact lens users [4]. As a well-studied organism, *P. aeruginosa* can also serve as a model for developing our understanding of anti-biofilm techniques.

To combat pathogenic biofilms one must either stop the initial attachment and growth of cells on the surface in question or be able to destroy the biofilm after it has matured. Biofilms can be resistant to penetration by antimicrobial chemicals [5] or the biocides undergo degradation through enzymes present in the EPS as they penetrate the film rendering them useless for disrupting the biofilm [6]. Phenotypic adaptation by cells within a biofilm can also render them resistant to biocides, even if those chemicals can penetrate the film [7]. The more realistic strategy for combatting biofilms is to prevent or slow down the attachment of cells to the surface.

This study is concerned with testing a new class of polymeric coatings that show promise in preventing the growth of biofilms on a surface. The coating is based on a macroporous poly(butyl methacrylate-co-ethylene dimethacrylate) (BMA-EDMA) polymer infused with the slippery lubricant perflouropolyether (PFPE) creating a surface coating called slippery BMA-EDMA [8]. The test is done by measuring the growth rate in a high shear stress fluid environment provided by a CDC bioreactor [9]. To gain some insight into the surface characteristics that promote or deter biofilm formation we compare the slippery BMA-EDMA growth rate with those on polycarbonate and borosilicate glass surfaces.

## Materials and Methods

### Bacteria culture

The PA01 strain of *P. aeruginosa* grown in a tryptic soy broth medium (BD^™^ Bacto^™^ Tryptic Soy Broth, Fisher Scientific, USA) was used for this study. Overnight cultures of PA01 in TSB grown at 37°C and shaken at 180 rpm were used to inoculate the bioreactor using 2 mL of suspended cell culture at ~10^8^ CFU/mL. All overnight cultures were inoculated from slants that are passaged no more than three times from frozen stock.

### Surface preparation and characterization

Polycarbonate and borosilicate glass coupons engineered for use in the CDC bioreactor (Model CBR 90-2, BioSurface Technologies Corporation, Bozeman, MT, USA) were obtained from BioSurface Technologies (RD 128-PC, RD 128-GL, BioSurface Technologies).

Coupons with the slippery BMA-EDMA were prepared locally according to the procedure described below. Before each experimental run with the CDC reactor, the polycarbonate and glass coupons were cleaned using the protocol described in Gores [9].

The slippery BMA-EDMA was assembled directly on glass coupons according to the methods outlined in Li et al. [8] and Levkin et al. [10] with a few modifications. First, borosilicate coupons were activated by immersion in 1 M NaOH for 1 h, immersion in 0.2 M HCl for 30 min, followed by washing with distilled water and drying with nitrogen gas. The activated coupons were then functionalized with a few drops of 20% 3-(trimethoxysilyl)propyl methacrylate [TMPMA, A17714 Alfa Aesar, Ward Hill, MA, USA] in ethanol for two 30 minute segments with reapplication of the solution after the first half hour. The functionalized coupons were then washed with acetone, dried with nitrogen gas, and placed in a custom polydimethylsiloxane [PDMS, 184 SIL ELAST KIT 0.5KG, Ellsworth Adhesives, Germantown, WI, USA] holder. The monomer solution was injected between the PDMS holder and coupons sandwiched against a glass slide [Glass B, 1025087, Schott Nexterion, Tempe, AZ, USA]. The monomer solution consisted of 24% wt Butyl methacrylate [BMA, M0081, TCI Chemicals, Portland, OR, USA], 16% wt ethylene glycol methacrylate [EDMA, 44151, Alfa Aesar], 40% wt 1-decanol, 20% wt cyclohexanol and 1% wt, with respect to monomer and crosslinker, 2,2-Dimethoxy-2-phenyl-acetophenone [DMPAP, 196118, Sigma-Aldrich, St. Louis, MO, USA]. The molds were incubated under UV light [bulb 34-0007-01, stand K126974, UVP, Upland, CA, USA] for 3 h leaving an opaque porous polymer. The coupons were then removed from the glass sandwich, immersed overnight in methanol and dried with nitrogen gas. The pores were then revealed by application of adhesive tape removing a smooth layer of polymer which develops at the polymer-glass slide interface. Finally, the coupons were infused with perfluoropolyether [PFPE, MS-1010, FluoroExtreme, Miller-Stephenson Chemical Co, Morton Grove, IL, USA] by dropping the liquid on the surface and allowing the excess to run off while sitting at a 20° angle with the table.

The average surface roughness, skewness and kurtosis measurements on the three surface materials were obtained using a Keyence VK-X200K laser scanning microscope (Keyence Corporation, Itaska, IL USA) that is able to perform non-contact measurements using an optical technique. This instrument allows the coupon surfaces to be scanned directly in air environment with no additional sample preparation. After images were obtained, automated roughness measurements were performed with the associated VK-Analyzer software over the 700 μm × 500 μm area of the image.

The static water contact angle was measured for each surface material using a locally built apparatus based on a design by Larmour et al. [11]. The procedure involved pipetting a 5 μL drop of deionized water onto the surface, photographing the magnified drop in the locally built setup, transferring the image file to a computer and finally measuring the contact angle with image [12] using the contact angle plugin and the manual point procedure.

### Operation of CDC bioreactor

*Pseudomonas aeruginosa* (PA01) biofilms were grown in the commercial CDC Biofilm Reactor from BioSurface Technologies. There were eight rods total for the reactor. Each rod contained three coupons arranged in a vertical position; each coupon possessed a diameter of 1.27 cm. The reactor, tubing and blank rods were sterilized in a Hirayama HV-110 Autoclave. The sterile Tryptic Soy Broth (TSB) growth media (from BD Difco^™^ Dehydrated Culture Media) was prepared in two 10 L carboys and sterilized in an American Manual Autoclave. A culture made of 25 mL of TSB was inoculated with PA01 and allowed to grow overnight. In a sidearm flask containing 25 mL of TSB, 100 μL of the overnight culture was added and was grown until the OD reached 0.20. The OD was checked using a Spectronic 20 Spectrophotometer. The reactor was filled with approximately 600 mL of sterile TSB growth media; 2 mL of 0.20 OD culture was then added and allowed to grow in the shear environment for 24 h at 125 rpm and 37°C. After the 24 h, the media flow began. There was approximately 360 mL of working volume in the reactor. The flow rate was chosen to keep the residence time of the media less than 20 min during the Continuous Flow, Stirred Tank Reactor (CFSTR) mode. TSB was pumped through the reactor from the top and the waste was dispensed from the side. A filtered port provided air to the bacteria. Every 8–10 h a rod was pulled and replaced with a sterile blank rod. Each set of coupons from the rods went through crystal violet assay.

### Crystal violet assay for biofilm accumulation measurement

At each time point, the coupons in a single rod were removed and rinsed in sterile distilled water. The coupons were blotted dried on a paper towel biofilm side up. The coupon was placed, film side up, in a 24 well plate containing 370 uL of 0.01% CV in water solution for 15 min at room temperature (RT). The coupons were rinsed in distilled water, blotted dried, and put into a well containing 7 glass beads with the film side down. To the same well, 350 μL 95% ethanol was added and incubated for 30 min at RT. After incubation, 50 μL crystal violet infused ethanol was diluted with 100 μL 95% ethanol in a 96-well plate. This is repeated two more times for a total of three 1:3 dilutions per coupon. The optical densities of the solutions were measured using the Cary 50 UV-Vis Spectrometer microplate reader at 600 nm.

## Results

The shear stress on the CDC bioreactor coupons can be estimated by assuming that the media behaves as a uniformly flowing Newtonian fluid. For this situation, the shear stress τ is related to the dynamic viscosity η and the fluid velocity gradient dv/dy by Equation (1) [13].

$$\tau =\eta \frac{dv}{dy}$$

We assume the viscosity of the fluid is close to that of water at 40°C, which, from the CRC Handbook of Chemistry and Physics, is 653.2 Pa.s. The velocity gradient is estimated by assuming the fluid has the velocity of the rotating reactor paddle at its edge and a velocity of zero at the surface of the coupon. The reactor paddle edge is 2.5 cm from the rotation axis and the coupon surface is 0.75 cm from the paddle edge. The angular speed of 125 rpm, which is 13.1 radians/s, then gives a fluid speed of 0.295 m/s at the paddle edge, so that the velocity gradient is 39.3 s^−1^. Using these estimates for dynamic viscosity and velocity gradient in Equation (1) gives a value of 0.0257 Pa or 0.257 dyne/cm^2^, for the shear stress.

**Figure 1:**
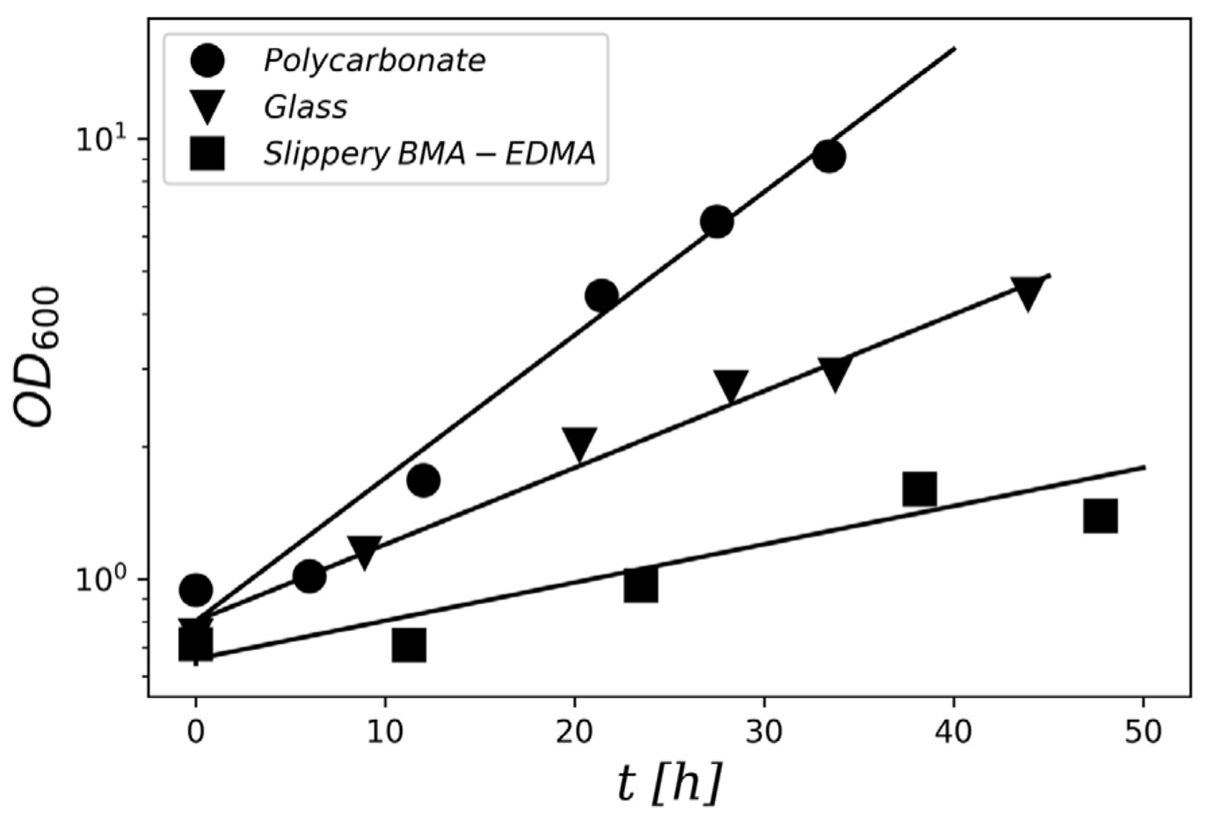
Growth curve of PA01 biofilm on polycarbonate, glass, and Slippery BMA-EDMA coupons. Relative biofilm accumulation measured using the OD_600_ from crystal violet assay as a function of time. A semi-log scale is used. The solid lines show fits of the data to an exponential model. The data point size indicates the approximate standard error associated with each measurement.

Biofilm growth on all three surface materials, glass, polycarbonate, and slippery BMA-EDMA, showed a well-defined period of exponential growth after the incubation period. Figure 1 shows an example of the growth curve observed on coupons of each surface material. Growth rates for each surface type were determined from such plots by fitting the crystal violet OD600 measurements to the exponential model of Equation (2) (Table 1).

$${OD}_{600}(t)={OD}_{600}(O)e^{\mu t}$$

To test whether the mean growth rates differ between surfaces, we calculated p-values for the hypothesis that compared means are the same using the Student’s t-test. If mean growth rates for different surfaces show statistically significant differences then the p-value should be close to zero. Table 2 gives the calculated p-values for comparing the mean growth rates for the three surfaces. The p-values for all surface comparisons are close to zero, although the value for the glass and slippery BMA-EDMA growth rates is a little higher than is traditionally accepted for assuming a statistically significant difference.

To explore the question of why there might be a difference in growth rates between the three surfaces we looked at the contact angle of water on each surface, as a measure of hydrophobicity, and at a measures of surface morphology, i.e. average surface roughness, skewness and kurtosis, performed with the Keyence VK-X100 microscope and VK Analyzer software. Table 3 summarizes these measurements.

## Discussion

Previous study of the slippery BMA-EDMA surface showed that it could reduce *P. aeruginosa* biofilm growth under low shear stress conditions, although the inhibitory effect depended on the strain of bacteria [8]. In this study we have extended understanding of biofilm growth on this material to include higher rates of shear stress on the surface. The reduction in growth rate on the slippery BMA-EDMA surface compared to polycarbonate and glass surfaces shown in Table 1 confirms the potential of this material for anti-biofouling use on biomedical devices.

**Figure 2:**
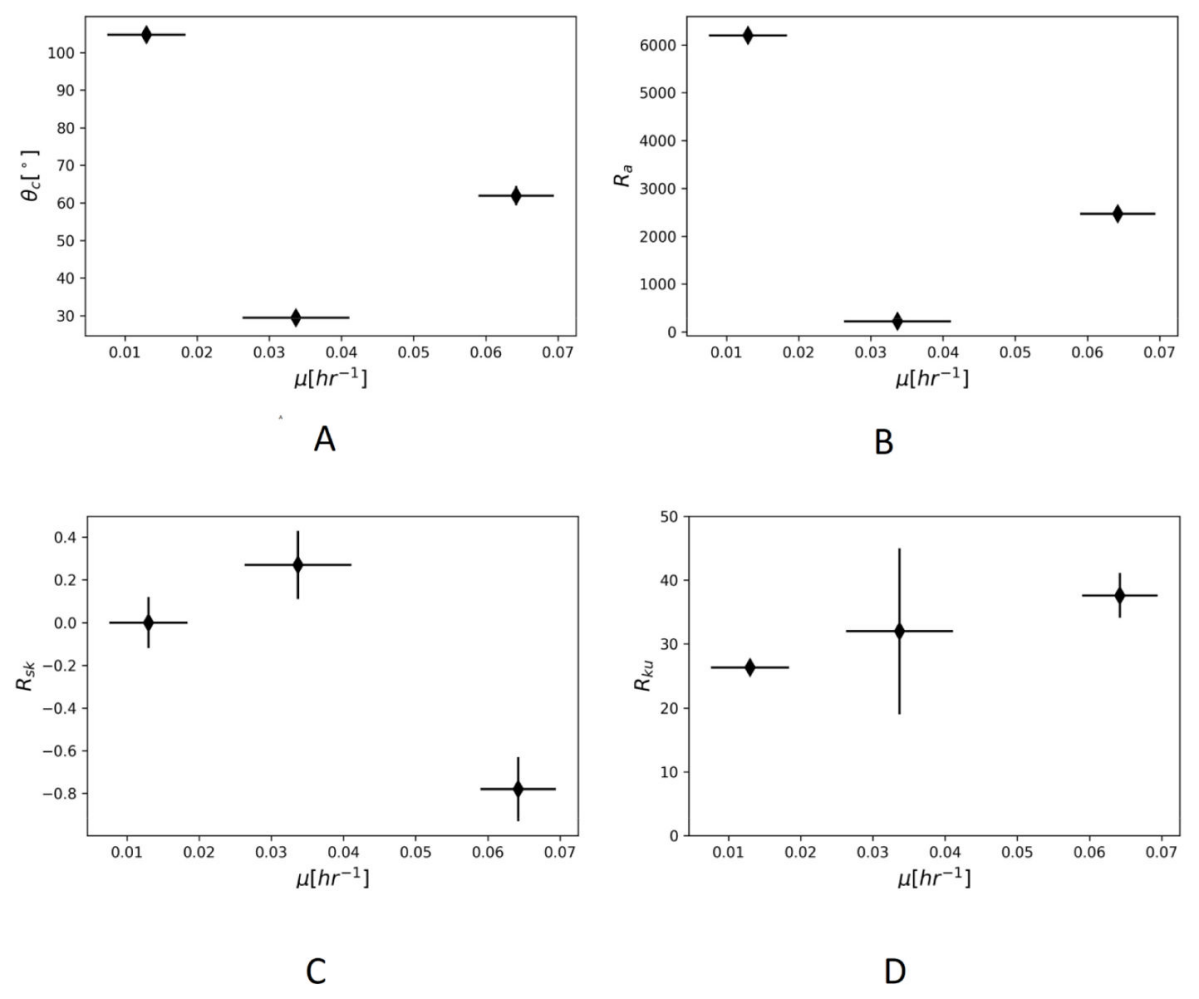
Surface characterization measurements as function of biofilm growth rate for slippery BMA-EDMA, glass, and polycarbonate surfaces. (A) Contact angle, (B) average surface roughness, (C) average surface skewness, and (D) average surface kurtosis.

The reason for growth reduction on the slippery BMA-EDMA surface remains an open question, but the surface characterization measurements do contain some possible directions to explore. The PFPE lubricant itself does not show antimicrobial properties [8], so the cause must lie with physical and chemical properties of the surface. Figure 2A suggests only a weak relationship, if any, between hydrophobicity of the surface and the biofilm growth rate. The correlation coefficient between growth rate and contact angle is −0.47. This observation agrees with many previous studies that show little correlation between biofilm growth and surface hydrophobicity [14,15].

Figure 2B also suggests only a very weak, if any, relationship between average surface roughness and growth rate. The correlation coefficient between growth rate and average surface roughness is −0.53. Figure 2C shows a stronger relationship between surface skewness and growth, with a correlation coefficient of −0.88. Figure 2D shows a strong relationship between surface kurtosis and growth rate, with a correlation coefficient of 0.96.

Surface profile skewness measures the asymmetry in the surface height distribution. Positive values occur when the distribution is skewed above the mean surface level, which occurs when there are many peaks and not many troughs. Negative values occur when the distribution is skewed below the mean surface level, which means there are many deep troughs and not many peaks. Surface profile kurtosis measures the sharpness of the surface profile distribution. Higher surface kurtosis values indicate more peaks in a surface region [16]. A similar trend was observed by Truong et al on titanium (Ti) surfaces noting that increased skewness and kurtosis on ECAP Ti correlated with greater retention of *Pseudomonas aeruginosa* and *Staphylococci aureus* [14]. Singh et al. observed on nanosized titanium oxide coated surfaces an increase in protein adhesion correlating with increasing skewness and kurtosis [17]. However, they also observed a significant decrease in *Staphylococci aureus* attachment on those same surfaces. Few studies to date have noted these parameters when quantifying biofilm growth experiments making these measurements potentially interesting to note in future studies to better understand the role of topography in biofilm growth.

## Conclusion

In conclusion, this study confirmed the significant reduction in biofilm growth rate on the slippery BMA-EDMA surface for the PA01 strain of *Pseudomonas aeruginosa* in a rich medium for higher shear stress fluid flow conditions compared to previous studies. We compared growth rates of the biofilm on the slippery BMA-EDMA, polycarbonate, and glass surface materials. We found only a weak correlation between surface hydrophobicity or average surface roughness and growth rate. We found a strong correlation between average surface skewness and kurtosis and growth rate. *Pseuodomonas aeruginosa* is a gram negative, rod shaped bacteria. As bacteria charge and shape can influence surface interactions more studies with a variety of bacteria would be informative to create a full picture of the anti-biofilm potential of the Slippery BMA-EDMA surface. However, the current study highlights previously unexplored topographical measurements, which may be insightful to include in further study of this and other anti-biofouling materials.

## Figures and Tables

**Table 1:** Growth rates of biofilm on the three surface materials. Growth rates μ and statistical uncertainty Δμ measured from fits of biofilm accumulation data to an exponential model.

Surface	μ hr^−1^	Δμ hr^−1^
Polycarbonate	0.0642	0.0052
Glass	0.0337	0.0074
Slippery BMA-EDMA	0.013	0.0054

**Table 2:** p-values from Student’s t-test for testing whether growth rate means are the same.

	Polycarbonate	Glass	Slippery BMA-EDMA
**Polycarbonate**	1	0.032	0.0027
**Glass**		1	0.092
**Slippery BMA-EDMA**			1

**Table 3:** Surface characterization measurements. ⊝ _c_ is the water contact angle. R_a_ is the average surface roughness. R_sk_ is average surface skewness. R_ku_ is average surface kurtosis.

Surface	⊝_c_	R_a_	R_sk_	R_ku_
**Polycarbonate**	61.9 ± 2.6	2472 ± 24	−0.78 ± 0.15	37.6 ± 3.5
**Glass**	29.41 ± 0.94	224.5 ± 1.5	0.27 ± 0.16	32 ± 13
**Slippery BMA-EDMA**	104.8 ± 1.0	6,193 ± 97	0.00 ± 0.12	26.30 ± 0.13
